# Pleiotropic Effects of c-di-GMP Content in *Pseudomonas syringae*

**DOI:** 10.1128/AEM.00152-19

**Published:** 2019-05-02

**Authors:** Tingting Wang, Zhao Cai, Xiaolong Shao, Weitong Zhang, Yingpeng Xie, Yingchao Zhang, Canfeng Hua, Stephan C. Schuster, Liang Yang, Xin Deng

**Affiliations:** a Department of Biomedical Sciences, City University of Hong Kong, Kowloon Tong, Hong Kong; b Singapore Centre for Environmental Life Sciences Engineering (SCELSE), Nanyang Technological University, Singapore; c Key Laboratory of Molecular Microbiology and Technology Ministry of Education, TEDA Institute of Biological Sciences and Biotechnology, Nankai University, Tianjin, China; d School of Medicine, Southern University of Science and Technology (SUSTec), Shenzhen, Guangdong, China; e School of Biological Sciences, Nanyang Technological University, Singapore; University of Manchester

**Keywords:** *Pseudomonas syringae*, T3SS, c-di-GMP, pleiotropic effects

## Abstract

Although the ubiquitous bacterial secondary messenger cyclic diguanylate (c-di-GMP) has important cellular functions in a wide range of bacteria, its function in the model plant pathogen Pseudomonas syringae remains largely elusive. To this end, we overexpressed Escherichia coli diguanylate cyclase (YedQ) and phosphodiesterase (YhjH) in P. syringae, resulting in high and low *in vivo* levels of c-di-GMP, respectively. Via genome-wide RNA sequencing of these two strains, we found that c-di-GMP regulates (i) *fliN*, *fliE*, and *flhA*, which are associated with flagellar assembly; (ii) *alg8* and *alg44*, which are related to the exopolysaccharide biosynthesis pathway; (iii) *pvdE*, *pvdP*, and *pvsA*, which are associated with the siderophore biosynthesis pathway; and (iv) *sodA*, which encodes a superoxide dismutase. In particular, we identified three promoters that are sensitive to elevated levels of c-di-GMP and inserted them into luciferase-based reporters that respond effectively to the c-di-GMP levels in P. syringae; these promoters could be useful in the measurement of *in vivo* levels of c-di-GMP in real time. Further phenotypic assays validated the RNA sequencing (RNA-seq) results and confirmed the effect on c-di-GMP-associated pathways, such as repressing the type III secretion system (T3SS) and motility while inducing biofilm production, siderophore production, and oxidative stress resistance. Taken together, these results demonstrate that c-di-GMP regulates the virulence and stress response in P. syringae, which suggests that tuning its level could be a new strategy to protect plants from attacks by this pathogen.

**IMPORTANCE** The present work comprehensively analyzed the transcriptome and phenotypes that were regulated by c-di-GMP in P. syringae. Given that the majority of diguanylate cyclases and phosphodiesterases have not been characterized in P. syringae, this work provided a very useful database for the future study on regulatory mechanism (especially its relationship with T3SS) of c-di-GMP in P. syringae. In particular, we identified three promoters that were sensitive to elevated c-di-GMP levels and inserted them into luciferase-based reporters that effectively respond to intracellular levels of c-di-GMP in P. syringae, which could be used as an economic and efficient way to measure relative c-di-GMP levels *in vivo* in the future.

## Abstract (precis)

The present work comprehensively analyzed the transcriptome and phenotypes that were regulated by c-di-GMP in P. syringae. Given that the majority of diguanylate cyclases and phosphodiesterases have not been characterized in P. syringae, this work provided a very useful database for the future study on regulatory mechanism (especially its relationship with T3SS) of c-di-GMP in P. syringae. In particular, we identified three promoters that were sensitive to elevated c-di-GMP levels and inserted them into luciferase-based reporters that effectively respond to intracellular levels of c-di-GMP in P. syringae, which could be used as an economic and efficient way to measure relative c-di-GMP levels *in vivo* in the future.

## INTRODUCTION

The bacterial secondary messenger cyclic diguanylate (c-di-GMP) regulates multiple important functions, including the transition from a planktonic lifestyle to a biofilm lifestyle and the biosynthesis of exopolysaccharides in the extracellular matrix of biofilms in many bacterial species (1–5). c-di-GMP is catalyzed by diguanylate cyclases (DGCs) and degraded by phosphodiesterases (PDEs). These enzymes are found in many bacterial species, such as Escherichia coli, Salmonella enterica, Bacillus subtilis, Pseudomonas aeruginosa, and Clostridium difficile (4, 6–8).

In Pseudomonas syringae pv. tomato DC3000, BifA and Chp8 have been identified as a PDE and a DGC, respectively (9, 10). The overexpression of BifA reduces the c-di-GMP level *in vivo* and elevates the virulence of pathogens in the Pseudomonas genus, whereas a Δ*bifA* mutant strain shows an elevated c-di-GMP level and reduced necrosis and chlorotic symptoms during infections (10). Although BifA and Chp8 perform opposite functions, both affect virulence-related phenotypes such as motility and biofilm formation (9, 10). A high intracellular c-di-GMP level drastically inhibits the biosynthesis of flagellin and bacterial motility but enhances the formation of biofilm. In contrast, a low c-di-GMP level strengthens bacterial motility and attenuates biofilm production in many bacteria (2, 5, 11–14). In P. aeruginosa, c-di-GMP regulates bacterial motility by controlling the expression of FliA, the key regulator of flagellar synthesis (15).

A high intracellular level of c-di-GMP induces P. aeruginosa pyoverdine synthesis, which is dependent on exopolysaccharides and DGC (16–19). Under iron-replete conditions, P. aeruginosa produces the major siderophore, pyoverdine, for the uptake of iron and rescue of iron starvation (20, 21). In P. syringae, pyoverdine is regulated by PvdS (20, 22, 23) and is assembled by nonribosomal peptide synthetase (24). PvsA is also essential for the biosynthesis of pyoverdine in Pseudomonas fluorescens ATCC 17400 (21).

During infection in a host, c-di-GMP regulates bacterial resistance against oxidative stress, which is crucial for pathogens to survive the host immune response (25). For example, in Salmonella enterica, a low level of c-di-GMP decreases resistance against hydrogen peroxide (H_2_O_2_) (13). In P. aeruginosa, an increased c-di-GMP level confers greater resistance to H_2_O_2_ (25). Scavenger superoxide dismutases are expressed by pathogens to resist reactive oxygen species (ROS), which damage bacterial DNA, RNA, proteins, and the stability of cell membranes (26, 27). For instance, the superoxide dismutases SodA and SodB convert superoxide O^2−^ into H_2_O_2_, which is decomposed into O_2_ and H_2_O with the help of KatG, KatE, or the alkyl hydroperoxide reductase AhpC (28–30).

YedQ and YhjH, the DGC and PDE from E. coli, have been used to tune the c-di-GMP levels in Burkholderia cenocepacia (31), Comamonas testosteroni (32), Pseudomonas aeruginosa (17), Pantoea alhagi (33), and Pseudomonas putida (34) and to investigate the effects of c-di-GMP in these bacteria. Therefore, to explore the functions of c-di-GMP in P. syringae, we used plasmid pBBR1MCS to overexpress *yedQ* and *yhjH* in P. syringae. We hypothesized that c-di-GMP has a divergent effect on virulence and stress responses in P. syringae.

In this study, RNA sequencing (RNA-seq) was performed to identify the c-di-GMP-dependent regulon in P. syringae. Based on the RNA-seq results, luciferase-based reporters were constructed to efficiently measure the intracellular c-di-GMP level. Phenotypic assays were further used to demonstrate that c-di-GMP regulates many important biological pathways in P. syringae, such as regulation of the type III secretion system (T3SS), motility, biofilm production, siderophore production, and oxidative stress resistance.

## RESULTS

### Overexpression of YedQ increases the intracellular c-di-GMP level in P. syringae.

**FIG 1:**
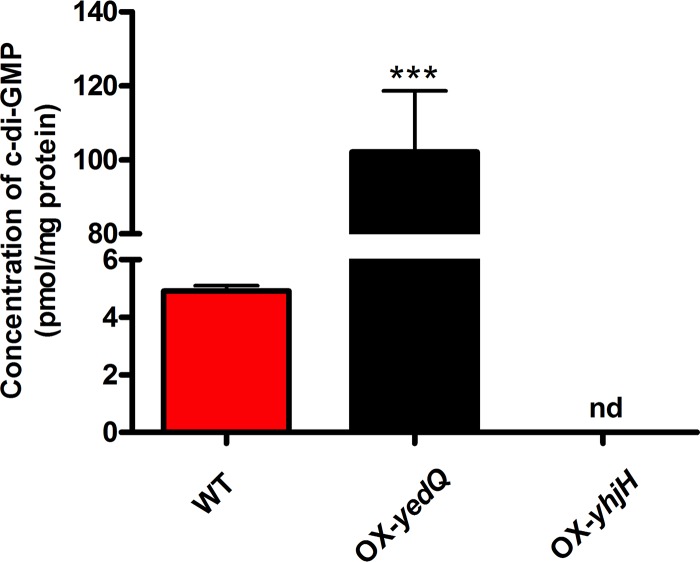
Quantification of high-c-di-GMP-content strain OX-*yedQ*, low-c-di-GMP-content strain OX-*yhjH*, and *P. syringae* pv. syringae B728a WT by mass spectroscopy analysis. nd, not determined. *, *P* < 0.05; ***, *P* < 0.001 by Student’s unpaired two-tailed *t* test compared to *P. syringae* pv. syringae B728a WT with equal variance. The OX-*yhjH* strain was under the lowest detection value. The experiment was repeated with three independent bacterial cultures.

To modulate the intracellular c-di-GMP level in P. syringae, OX-*yedQ* and OX-*yhjH* strains were generated via the overexpression of YedQ and YhjH, which function as c-di-GMP synthase and phosphodiesterase in P. syringae pv. syringae B728a, respectively. The c-di-GMP concentrations of wild-type, OX-*yedQ*, and OX-*yhjH* strains were quantified by liquid chromatography-mass spectrometry (LC-MS) at the stationary-growth phase. As expected, the c-di-GMP concentration in the OX-*yedQ* strain (102.12 pmol/mg) was more than 20 times greater than that in the wild type (4.91 pmol/mg; *P* = 0.00026) (Fig. 1), which demonstrates that the overexpression of the OX-*yedQ* strain leads to the elevated production of intracellular c-di-GMP in P. syringae. In contrast, the level of c-di-GMP in the OX-*yhjH* strain was below the detection limit of LC-MS (Fig. 1), which suggests that overexpression of YhjH results in the degradation of c-di-GMP in P. syringae. Therefore, the heterogeneous expression of YedQ in P. syringae is effective in generating a high level of c-di-GMP.

### c-di-GMP-dependent regulon in P. syringae.

**FIG 2:**
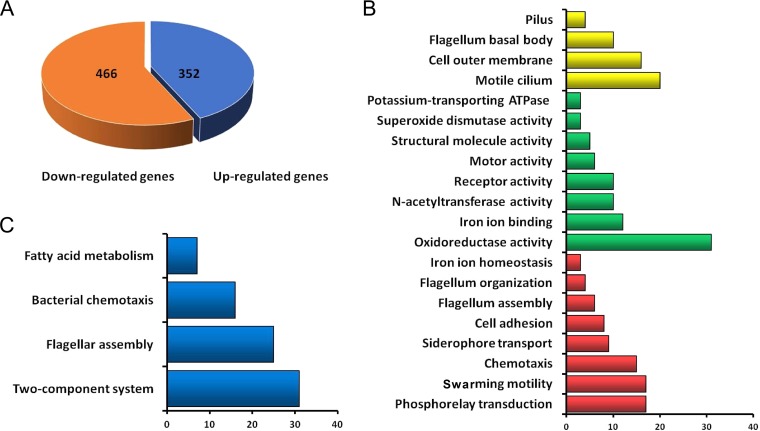
RNA-seq analysis between OX-*yedQ* and OX-*yhjH* strains. (A) Pie chart of 818 DEGs significantly regulated by a high level of c-di-GMP. The number of downregulated DEGs is indicated in orange, and the number of upregulated DEGs is indicated in blue. All experiments were performed in triplicate. Error bars indicate standard deviations. (B) Gene Ontology enrichment analysis in “biological process,” “cellular component,” and “molecular function” categories for genes upregulated and downregulated in response to an elevated c-di-GMP level. In all, GO terms were overrepresented by >2-fold enrichment values, with a *P* value of <0.05. (C) Functional classification of KEGG pathway. The KEGG pathways were summarized in seven main categories for upregulated and downregulated genes, as follows: two-component system, flagellar assembly, bacterial chemotaxis, fatty acid metabolism, biotin metabolism, starch and sucrose metabolism, and tryptophan metabolism. The *x* axis indicates the numbers of genes of the KEGG metabolic pathways. The *y* axis indicates terms of KEGG metabolic pathways, including two items of upregulation and five items of downregulation. In all categories, the *P* value was <0.05.

To reveal the c-di-GMP-dependent regulons in P. syringae, RNA-seq was performed to compare the transcriptomic profiles of the OX-*yedQ* and OX-*yhjH* strains; 818 differentially expressed genes (DEGs) were identified between these two strains (Fig. 2A; see also Table S1 in the supplemental material). Of these DEGs, 352 were upregulated and 466 were downregulated in the OX-*yedQ* strain (*P* < 0.05; more than 2-fold enrichment; Fig. 2A). Enrichment analyses were performed on these DEGs via the Gene Ontology (GO) or the KEGG pathway. GO enrichment analysis highlighted the genes involved in flagellum-dependent cell motility, phosphorelay signal transduction, flagellum organization, siderophore transport, cell adhesion, and chemotaxis (Fig. 2B). Notably, the motility-related genes were significantly enriched (Fig. 2B). The genes associated with oxidoreductase activity and siderophore biosynthesis and transport were also enriched significantly (*P* < 0.05; Fig. 2B). According to KEGG analysis, genes associated with two-component systems were identified as the most enriched pathway (Fig. 2C). Flagellar assembly, fatty acid biosynthesis, and bacterial chemotaxis were also significantly enriched (Fig. 2C). Among the DEGs, Psyr_0610, Psyr_0685, and Psyr_5026 were selected for c-di-GMP-sensitive reporter construction. In sum, transcriptomic profiling analysis characterized the c-di-GMP-dependent regulon in P. syringae, thus indicating that c-di-GMP regulates multiple biological pathways, including those for flagellar motility, iron-binding siderophore, chemotaxis, and oxidoreductase activity. Furthermore, the RNA-seq assay was repeated by using the wild-type (WT) and OX-*yedQ* strains (Table S2). GO enrichment analysis highlighted the genes involved in flagellum-dependent cell motility, phosphorelay signal transduction, and bacterial chemotaxis (Table S3), which is consistent with the RNA-seq assay by using the OX-*yedQ* and OX-*yhjH* strains (Fig. 2 and Table S1).

### Luciferase-based reporters reflect the c-di-GMP level *in vivo*.

**FIG 3:**
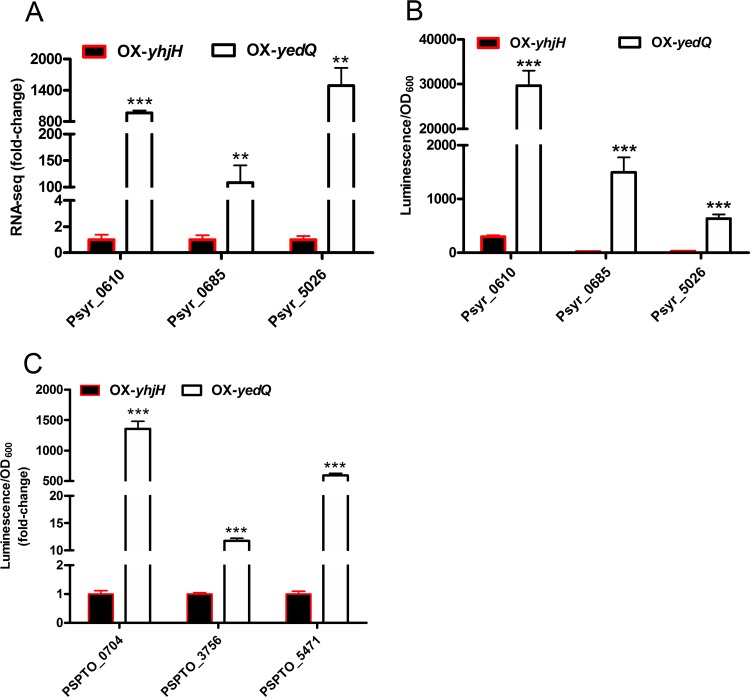
Five c-di-GMP-sensitive genes in *P. syringae*. (A) Fold change for five genes based on the RNA-seq results. OX-*yedQ* is the strain harboring pBBR1MCS5-*yedQ*. (B) Luminescence of strains with pMS402-*lux* reporters driven by promoters of Psyr_0610, Psyr_0685, and Psyr_5026. (C) Luminescence of strains with pMS402-*lux* reporters driven by promoters of PSPTO_0704, PSPTO_3756, and PSPTO_5471, cultivated for 12 h. *, significantly different from OX-*yhjH* strain at 12-h time point, *P* < 0.05; **, significantly different from OX-*yhjH* strain at 12-h time point, *P* < 0.01; ***, *P* < 0.001 at 12-h time point. All experiments were performed in triplicate. Error bars indicate standard deviations.

Mass spectrometry is widely used for the accurate quantification of the intracellular c-di-GMP concentration (35, 36). Here, we attempted to develop an economical and convenient method to measure the relative c-di-GMP concentrations in P. syringae using transcriptional fusion bioassays. We sought to make c-di-GMP biological reporters by fusing the promoters of c-di-GMP-induced genes to a promoterless luciferase gene. We first selected three top-induced candidates (Psyr_0610, Psyr_0685, and Psyr_5026) from our RNA-seq data sets (Fig. 3A). These three constructed plasmids were electrotransformed into the OX-*yedQ* and OX-*yhjH* strains to measure the luminescence value (in counts per second). All of the reporters (Psyr_0610, Psyr_0685, and Psyr_5026) showed significantly higher *lux* levels in the OX-*yedQ* strain than in the OX-*yhjH* strain (100-, 60-, and 20-fold, respectively; Fig. 3B), which indicates that these three promoters were very sensitive to the high levels of c-di-GMP in P. syringae pv. syringae B728a. In particular, the Psyr_0610 promoter was the most sensitive, with an induction of 100-fold, so it could be used as an efficient method to measure *in vivo* c-di-GMP levels (Fig. 3B).

To examine whether their homogenous genes in P. syringae pv. tomato DC3000 were also sensitive to the c-di-GMP level, we constructed their counterparts in P. syringae pv. tomato DC3000 (PSPTO_0704, PSPTO_3756, and PSPTO_5471). The resulting plasmids were electrotransformed into the OX-*yedQ* and OX-*yhjH* strains and showed even better results than their counterparts in P. syringae pv. syringae B728a. These promoters were induced ∼1,500-, 13-, and 500-fold, respectively, in the OX-*yedQ* strain (Fig. 3C). This luciferase-based assay also confirmed that overexpression of YedQ elevated the production of c-di-GMP in P. syringae. In sum, our newly constructed luciferase-based reporters can be used for sensitive monitoring of c-di-GMP levels *in vivo*.

### Expression of T3SS is suppressed by c-di-GMP.

**FIG 4:**
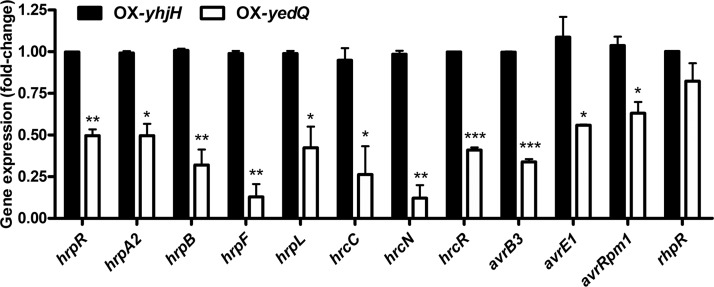
c-di-GMP negatively regulated T3SS in *P. syringae*. RT-qPCR of genes related to T3SS. The relative gene expression level in the OX-*yhjH* strain was set to 1, and the other values were adjusted accordingly. *, significantly different between OX-*yedQ* and OX-*yhjH* strains, *P* < 0.05; **, significantly different between OX-*yedQ* and OX-*yhjH* strains, *P* < 0.01; ***, significantly different between OX-*yedQ* and OX-*yhjH* strains, *P* < 0.001. All experiments were performed in duplicate. Error bars indicate standard deviations.

Studies have shown that c-di-GMP can modulate virulence and the T3SS in some plant pathogens (4, 8, 36). For example, a higher level of c-di-GMP leads to repression of the T3SS in P. aeruginosa (36). To explore the effects of c-di-GMP in P. syringae T3SS and virulence, we identified 12 T3SS genes from our RNA-seq data and verified them by using reverse transcription-quantitative PCR (RT-qPCR). As shown in Fig. 4, the expression of *hrpR*, *hrpL*, *hrpA2*, *hrpB*, *hrpF*, *hrcC*, *hrcN*, *hrcR*, *avrB3*, *avrE1*, and *avrRPM1* was suppressed by 2- to 3-fold in the OX-*yedQ* strain in minimal medium compared with that of the OX-*yhjH* strain, thus suggesting an inhibitory effect of c-di-GMP on the T3SS, which is consistent with the result for P. aeruginosa (36). We also tested whether the inhibition was mediated by RhpR, a known T3SS repressor (37, 38). However, the expression of *rhpR* showed no significant difference between the OX-*yedQ* and OX-*yhjH* strains (Fig. 4), which suggests that c-di-GMP inhibits the T3SS via factors other than RhpRS.

### c-di-GMP negatively controls motility by regulating the expression of *flhA, fliN*, and *fliE*.

**FIG 5:**
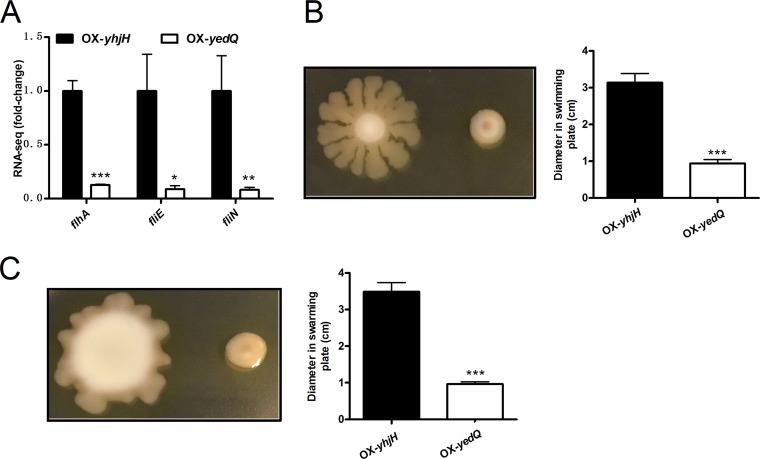
Motility is negatively regulated by c-di-GMP in *P. syringae*. (A) Fold change from RNA-seq result of genes related to flagellar motility. The relative gene expression level in the OX-*yhjH* strain was set to 1, and the other values were adjusted accordingly. (B) Swimming agar plate assay. Photos were taken after 36 h at 28°C. The swimming abilities of different strains were determined by the diameter of zone of motility, as shown in the bar graph. (C) Swarming agar plate assay. Phenotypic photos were taken after 72 h at 28°C. The swarming abilities of different strains were determined by the diameter of zone of motility, as shown in the bar graph. *, significantly different between OX-*yedQ* and OX-*yhjH* strains, *P* < 0.05; **, *P* < 0.01; ***, *P* < 0.001.

In other Pseudomonas species, motility was inhibited by strains with higher intracellular c-di-GMP levels (5, 12, 14). Our RNA-seq results showed that seven known operons (*flhAF*, *fliLMNPQR-flhB*, *fliEFJ*, *fliS-Psyr_3462*, *flgFGHIJKL*, *flgBCDE*, and *flgA*) associated with flagellar synthesis were significantly downregulated in the OX-*yedQ* strain compared with the OX-*yhjH* strain (Table S1 and Fig. 5A). In addition, the transcription levels of a group of hypothetical genes, such as Psyr_3466 (encoding flagellin; 4-fold less) and Psyr_3460 (encoding flagellar sensor histidine kinase FleS), were also repressed (12-fold less) by c-di-GMP (Table S1). The phenotypic experiments showed that the swarming motility and swimming motility of the OX-*yedQ* strain were significantly compromised (3-fold less) compared with those of the OX-*yhjH* strain (Fig. 5B and C). The elevated c-di-GMP level had significant inhibitory effects on the motility of P. syringae.

### c-di-GMP is required for biofilm formation in P. syringae.

**FIG 6:**
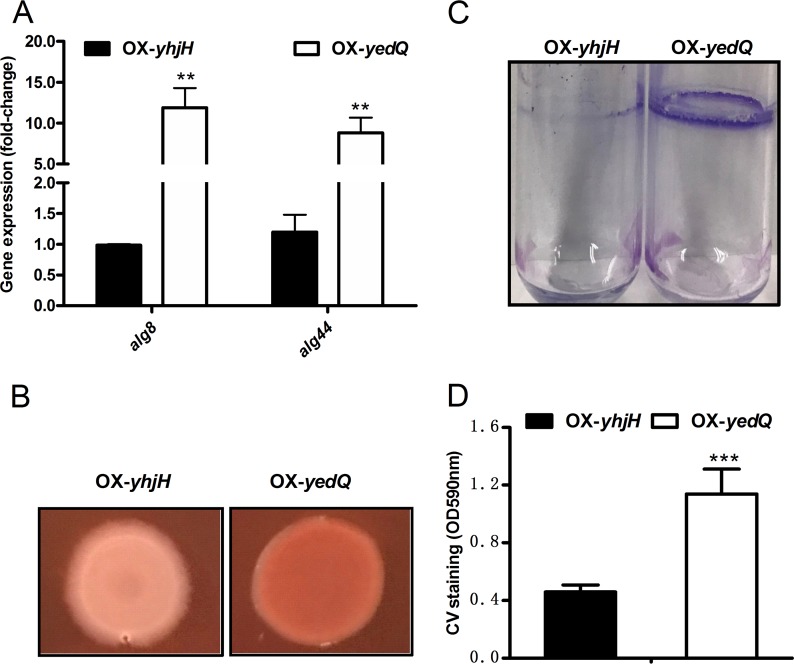
High c-di-GMP enhanced biofilm production. (A) RT-qPCR validation of *alg8* and *alg44* for the OX-*yedQ* strain compared to the OX-*yhjH* strain. The relative gene expression level in the OX-*yhjH* strain was set to 1, and the other values were adjusted accordingly. (B) Congo red phenotypic experiment of OX-*yedQ* and OX-*yhjH* strains. Photos were taken after 4 days of cultivation at 28°C. **, significantly different between OX-*yedQ* and OX-*yhjH* strains, *P* < 0.01; ***, significantly different between OX-*yedQ* and OX-*yhjH* strains, *P* < 0.001. Three Congo red plates were used for two strains, and the experiment was repeated with three independent bacterial cultures. (C) Crystal violet (CV) staining of OX-*yedQ* and OX-*yhjH* strains for quantifying relative biofilm biomass. Photo in the upper part illustrates biofilm formed at liquid-air interface by the two strains and stained by crystal violet in tubes. (D) The absorbance of crystal violet at 590 nm was measured. Error bars indicate standard deviations.

In P. aeruginosa, the overexpressing YhjH strain showed less biofilm formation than did the wild type (20). In E. coli, c-di-GMP positively regulates genes associated with motile-sessile transition and biosynthesis and with the secretion of exopolysaccharides (EPS) in biofilms (3, 4). Here, we speculated that c-di-GMP has similar functions in P. syringae. As expected, the expression of *alg8* and *alg44*, which are involved in EPS biosynthesis, was increased by 3- to 4-fold in the OX-*yedQ* strain based on RT-qPCR (Fig. 6A). To further verify this regulation, the Congo red assay and quantitative detection of biofilms were performed in both strains. As shown in Fig. 6B, EPS production was greater in the OX-*yedQ* strain (more mucoid colonies) than in the OX-*yhjH* strain. Biofilm formation was also much higher in the OX-*yedQ* strain, as shown in Fig. 6C and D. These results indicate that c-di-GMP positively regulates biofilm formation in P. syringae.

### c-di-GMP positively regulates siderophore production.

**FIG 7:**
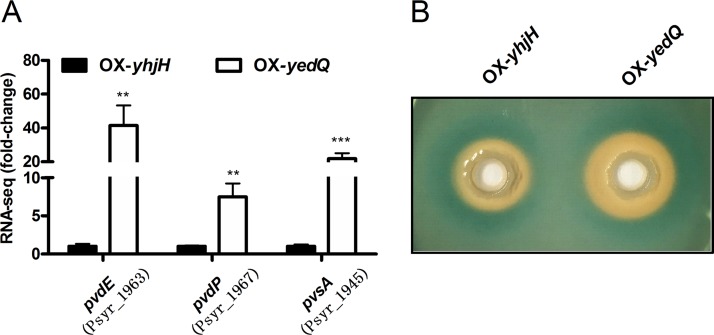
OX-*yedQ* strain produced excess pyoverdine. (A) The expression fold change of *pvdP*, *pvdE*, and *pvsA* based on RNA-seq data. The relative gene expression level in the OX-*yhjH* strain was set to 1, and the other values were adjusted accordingly. (B) CAS agar plate experiment. The CAS plate was photographed after 48 h of cultivation with white background. **, significantly different between OX-*yedQ* and OX-*yhjH* strains, *P* < 0.01; ***, significantly different between OX-*yedQ* and OX-*yhjH* strains, *P* < 0.001. Three CAS plates were used for two strains, and the experiment was repeated with three independent bacterial cultures. Error bars indicate standard deviations.

Of the DEGs in our RNA-seq data, genes catalogued as “iron ion binding” or as “siderophore transport” were significantly enriched in gene ontology analysis (Fig. 2B). The expression levels of pyoverdine transporter PvdE (32) and peptide synthase PvdP and PvsA (30, 31) were upregulated 40-, 7-, and 20-fold, respectively (Fig. 7A). Given that pyoverdine was characterized by siderophore production, iron acquisition, virulence, and growth under iron-restricted conditions (24), we further tested whether a high c-di-GMP content could induce siderophore production in P. syringae. In a chrome azurol S (CAS)-based iron uptake assay, the OX-*yedQ* strain exhibited a larger orange halo (more than 2-fold) than the OX-*yhjH* strain (Fig. 7B), which is consistent with a previous study in P. syringae pv. phaseolicola 1448A (24). c-di-GMP positively regulates the biosynthesis, assembly, and transport of siderophores in P. syringae.

### c-di-GMP positively regulates resistance to oxidative stress.

**FIG 8:**
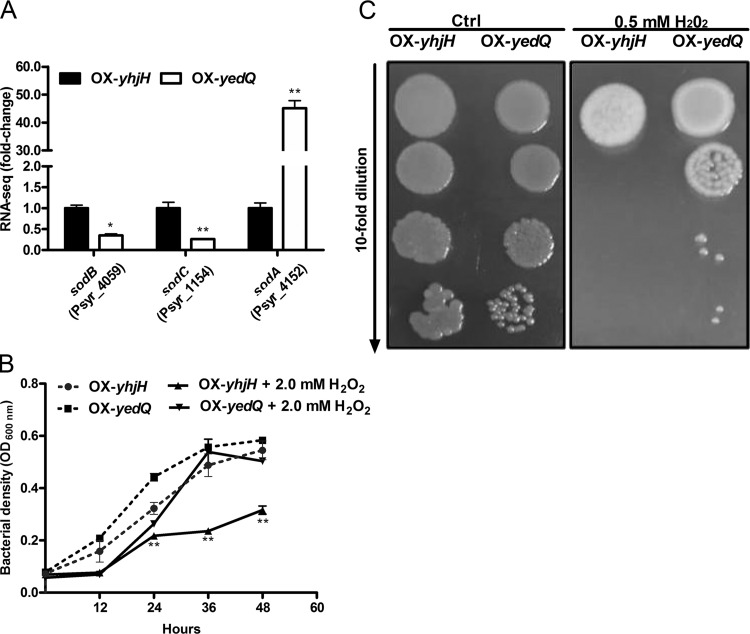
OX-*yedQ* strain was more resistant to H_2_O_2_ in *P. syringae*. (A) The expression of *sodA*, *sodB*, and *sodC* based on RNA-seq. The relative gene expression level in the OX-*yhjH* strain was set to 1, and the other values were adjusted accordingly. (B) OD_600_ of bacterial culture in 2 mM H_2_O_2_ in KB liquid medium after 48 h of cultivation. The OD_600_ was measured for every 12 h. **, significantly different between OX-*yedQ* and OX-*yhjH* strains, *P* < 0.01; ***, significantly different between OX-*yedQ* and OX-*yhjH* strains, *P* < 0.001. All experiments were performed in triplicate. Error bars indicate standard deviations. (C) Hydrogen peroxide resistance of colony assay. Bacterial culture was adjusted to an OD_600_ of ∼1.0. A KB agar plate was added 0.5 mM H_2_O_2_ and was dotted with 10 μl of 10-fold-diluted bacteria. Photos were taken after 3 days of cultivation at 28°C. Three H_2_O_2_ plates were used for two strains, and the experiment was repeated with two independent bacterial cultures. Ctrl, control.

Cytotoxic ROS, such as the superoxide radical O_2_^–^, H_2_O_2_, and hydroxyl radicals, can damage DNA, proteins, and lipids, resulting in a toxic effect on pathogenic bacteria (26). To protect bacteria from the ROS produced by plant cells during infection, it is important for pathogenic bacteria to inactivate ROS with their antioxidant enzymes, such as superoxide dismutase. Based on our RNA-seq data, genes that encode superoxide dismutase, such as *sodA*, were differentially expressed. Notably, the RNA-seq data showed that the expression of *sodA* was induced about 40-fold in the OX-*yedQ* strain, while the expression of *sodB* and *sodC* was repressed (Fig. 8A). We hypothesized that c-di-GMP mediates the resistance to oxidative stress in P. syringae. To test the phenotypes of the elevated c-di-GMP level, H_2_O_2_ tolerance tests were performed in liquid and solid KB media for the OX-*yhjH* and OX-*yedQ* strains. The OX-*yhjH* strain showed no growth in 6 mM H_2_O_2_, but the OX-*yedQ* strain grew well in 10 mM H_2_O_2_ for 18 h (data not shown). The OX-*yedQ* strain grew better in 2 mM H_2_O_2_ than the OX-*yhjH* strain (Fig. 8B). The result of the plate assay for H_2_O_2_ resistance also demonstrated that the OX-*yedQ* strain showed stronger tolerance against H_2_O_2_ than did the OX-*yhjH* strain (Fig. 8C). Taken together, the results suggest that c-di-GMP positively regulates resistance to oxidative stress by inducing the transcription of *sodA*.

## DISCUSSION

**FIG 9:**
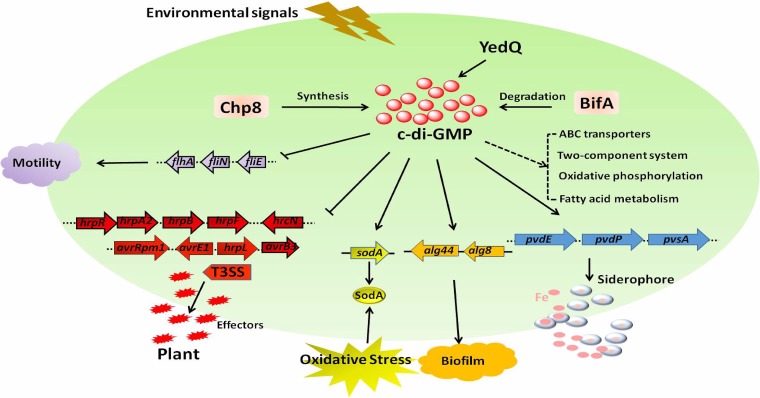
Schematic of pleiotropic effects of c-di-GMP in *P. syringae*. Overexpressed YedQ increased the c-di-GMP content in *P. syringae*. Enhanced c-di-GMP level positively controlled biofilm formation, oxidative stress resistance, and siderophore production but negatively regulated motility and T3SS. Enhanced c-di-GMP also regulated ABC transporters, two-component systems, oxidative phosphorylation, and fatty acid metabolism. Solid black arrows indicate positive regulation, solid line T-bars present negative regulation, and dashed line represents direct or indirect influence.

The overexpression of E. coli YedQ and YhjH protein is widely used to study the function of c-di-GMP in many Pseudomonas species (34, 39). The results of mass spectroscopy confirmed that the exogenous YedQ and YhjH enabled P. syringae to alter the c-di-GMP level (Fig. 9). The concentration of c-di-GMP was below the detection limit in the OX-*yhjH* strain. The three most sensitive c-di-GMP-responsive reporters (Psyr_0610-*lux*, Psyr_0685-*lux*, and Psyr_5026-*lux*) were identified and constructed and can be used to measure c-di-GMP levels *in vivo*. We were intrigued to find that the promoter of exogenous gene PSPTO_0704 of P. syringae pv. tomato DC3000 showed the highest induction level (1,000-fold) between the two strains. In P. aeruginosa, the c-di-GMP-responsive reporter *brlR-lux* was induced 100-fold by a high c-di-GMP level (40). The differences in the fold changes between PSPTO_0704-*lux* and *brlR-lux* may have been caused by the differences in the intracellular c-di-GMP levels in the two Pseudomonas strains.

The downregulation of a group of T3SS genes, including *hrpR*, *hrpA2*, *hrpB*, *hrpF*, *hrpL*, *hrcC*, *hrcN*, *hrcR*, *avrB3*, *avrE1*, and *avrRPM1* in the OX-*yedQ* strain, with a high c-di-GMP level, suggests that c-di-GMP negatively regulates T3SS in P. syringae. However, the underlying mechanism requires further study. Studies have shown that c-di-GMP inhibits T3SS but activates the type VI secretion system (T6SS) in different bacteria (36, 41, 42). Similarly, T3SS genes were regulated by c-di-GMP in P. syringae (Table S1).

Jenal et al. showed that c-di-GMP regulates bacterial lifestyles, including swimming, swarming, and biofilm formation, to alter bacterial virulence in P. aeruginosa (4). Chp8 elevates EPS production and negatively regulates swarming motility, whereas BifA negatively regulates swarming motility and positively regulates swimming motility in P. syringae pv. tomato DC3000 (9, 10). In P. aeruginosa PA14 and P. putida KT2442, BifA only affects swarming motility (43). In this study, an elevated level of c-di-GMP suppressed both swimming and swarming motility in P. syringae (Fig. 5B and C). The differences between P. syringae pv. syringae B728a and other Pseudomonas strains may have resulted from other functions of Chp8 and BifA. Biofilm production was positively regulated by c-di-GMP in P. syringae, which is consistent with the results for other bacterial species (4, 8). Furthermore, our study showed that c-di-GMP elevated the expression of lipopolysaccharide (LPS)-related genes. The expression of Psyr_0610 (encoding O-antigen ABC transporter and permease protein) and Psyr_0612 (encoding lipopolysaccharide biosynthesis protein) was 100-fold higher in the OX-*yedQ* strain than in the OX-*yhjH* strain, which suggests that c-di-GMP regulates biofilm formation by elevating the production of LPS in P. syringae.

Pyoverdine production and oxidative stress resistance are regulated by c-di-GMP in P. syringae (25, 44). Pseudomonas species synthesize pyoverdine, the key virulence factor, to take up iron from the extracellular medium to rescue iron starvation (44). c-di-GMP positively controls the siderophore pyoverdine to acquire iron from iron-replete medium (16). In addition to *pvdE*, *pvdP*, and *pvsA*, five genes that encode the putative siderophore nonribosomal peptide synthase (Psyr_1956, Psyr_1957, Psyr_1958, Psyr_1959, and Psyr_1960) were significantly upregulated in strains that overexpressed c-di-GMP (Table S1), which suggests that c-di-GMP regulates the uptake of iron by inducing the synthesis, export, and assembly of pyoverdine in P. syringae. c-di-GMP regulates the oxidative stress resistance in many bacterial strains (13, 28, 45), but the mechanism remains largely unclear. These results indicate that SodA, but not SodB or SodC, plays a major role in the c-di-GMP-mediated response against oxidative stress.

Taken together, these results demonstrate that the pleiotropic molecule c-di-GMP globally regulates many important intracellular activities and behaviors in P. syringae (Fig. 9). In particular, c-di-GMP inhibits motility and T3SS and induces biofilm formation, pyoverdine production, and oxidative stress resistance in P. syringae. The newly constructed P. syringae-specific *lux* reporters provide an economical and effective method to detect c-di-GMP levels *in vivo*. We propose that tuning the c-di-GMP level offers a new strategy to protect plants from attacks by P. syringae.

## MATERIALS AND METHODS

### Strains, plasmids, primers, and growth conditions.

The plasmids, bacterial strains, and primers used in this study are listed in Table 1. Unless otherwise indicated, Pseudomonas syringae pv. syringae B728a and its derivatives were grown in KB medium, consisting of 20 g proteose peptone, 1.5 g anhydrous K_2_HPO_4_, 15 ml glycerol, and 1.5 g MgSO_4_ per liter, with or without agar at 28°C or shaking at 250 rpm. The concentration and categories of antibiotics were added as follows: for P. syringae pv. syringae B728a wild-type (WT) strain, 25 μg/ml rifampin in KB agar medium; for the *yedQ*-overexpressing strain (OX-*yedQ*), 25 μg/ml rifampin and an additional 60 μg/ml gentamicin in KB agar medium but 30 μg/ml gentamicin in KB liquid medium; for the *yhjH*-overexpressing strain (OX-*yhjH*) strain, 25 μg/ml rifampin and an additional 60 μg/ml tetracycline; and for the strains containing the pMS402 plasmid, 100 μg/ml kanamycin was added, while in E. coli, 50 μg/ml kanamycin was added. Experiments for OX-*yedQ* and OX-*yhjH* construction, RNA-seq, and liquid chromatography mass spectrometry (LC-MS) quantification of c-di-GMP were performed at Nanyang Technological University in Singapore, and other experiments were finished at the Department of Biomedical Sciences, City University of Hong Kong, Kowloon Tong, Hong Kong.

**TABLE 1:** Strains, plasmids, and primers used in this study

Strain, plasmid, or primer	Description or sequence (5ʹ–3ʹ)^*a*^	Reference or application
Strains		
*Pseudomonas syringae* pv. syringae B728a	Prototypic wild-type strain, Rif^r^	54
OX-*yedQ* mutant	*P. syringae* pv. syringae B728a containing pBBR1MCS5-*yedQ*, Rif^r^	This study
OX-*yhjH* mutant	*P. syringae* pv. syringae B728a containing pBBR1MCS3-*yhjH*, Rif^r^	This study
Plasmids		
pBBR1MCS-5	Overexpression vector, Gm^r^	55
pBBR1MCS-3	Overexpression vector, Tc^r^	55
pBBR1MCS5-*yedQ*	Overexpresses YedQ under *lac* promoter (HindIII/BamHI), *yedQ* gene is cloned from pYedQ plasmid vector, Gm^r^	56
pBBR1MCS3-*yhjH*	Overexpresses YhjH under *lac* promoter (HindIII/BamHI), *yhjH* gene is cloned from pYhjH plasmid vector, Gm^r^	34
pMS402	Expression reporter plasmid carrying promoterless *luxCDABE*; Kn^r^	46
pMS402-0610	*lux* reporter fused with promoter of Psyr_0610. Kn^r^	This study
pMS402-0685	*lux* reporter fused with promoter of Psyr_0685, Kn^r^	This study
pMS402-1131	*lux* reporter fused with promoter of PSPTO_1131, Kn^r^	This study
pMS402-3767	*lux* reporter fused with promoter of PSPTO_3767, Kn^r^	This study
pMS402-5026	*lux* reporter fused with promoter of PSPTO_5026, Kn^r^	This study
Primers		
Psyr0610BamHI-F	TCGTCTTCACCTCGAGGGGATCCGAGGAGCCTCGCTTGTTCAAG	Reporter construction
Psyr0610BamHI-R	GCGGCCGCAACTAGAGGATCCTAATGAGAAAATCAGAGAGAG	Reporter construction
Psyr0685BamHI-F	TCGTCTTCACCTCGAGGGGATCCTCATCGCTCCTGCGTTTG	Reporter construction
Psyr0685BamHI-R	GCGGCCGCAACTAGAGGATCCTGCATGCCGTCCTTGCGTC	Reporter construction
Psyr5026BamHI-F	TCGTCTTCACCTCGAGGGGATCCCACCCTGTTGTGCGCCTCG	Reporter construction
Psyr5026BamHI-R	GCGGCCGCAACTAGAGGATCCTCGAGATTTCCCAAGAGT	Reporter construction
hopAH2-F	AGGACCTGAAAGCGATTGGA	RT-qPCR validation
hopAH2-R	GAGCTTATCCAACTGCCTGC	RT-qPCR validation
AvrE1-F	CATAGCAACTCCACAGCGAC	RT-qPCR validation
AvrE1-R	TCATCAATGGTCACGTTCGC	RT-qPCR validation
HrpA2-F	CAGGGCATCAACAGCGTA	RT-qPCR validation
hrpA2-R	GTCGATACTGTCAGTGCTGC	RT-qPCR validation
hrpB-F	GTCGATGAAGAAAGCCTCCG	RT-qPCR validation
hrpB-R	CAGTCTTGCTCACCACCTTG	RT-qPCR validation
hrpF-F	TAACCTCGATTCCACGCTCA	RT-qPCR validation
hrpF-R	CCTCACTGAAGGCATCGATG	RT-qPCR validation
hrpL-F	GTGTTTCTCGAGGCGTTACG	RT-qPCR validation
hrpL-R	CTGGCGATACATTTTGCGGA	RT-qPCR validation
hrcC-F	CTTCACGCAGATGGTCGATG	RT-qPCR validation
hrcC-R	CACAGGCTGTCGGTTTCA	RT-qPCR validation
hrcN-F	GGCCGCCTATAAACAAGTG	RT-qPCR validation
hrcN-R	GGAACGCGTTTATAGCCTCG	RT-qPCR validation
hrcR-F	GCAGCCTCAAAGTCGTCATC	RT-qPCR validation
hrcR-R	CATGCGCTGGGTATTTTCCA	RT-qPCR validation
avrB3-F	TCTCCCACACAGCAATACGT	RT-qPCR validation
avrB3-R	GGATCCTTTGTTCTGTCGGC	RT-qPCR validation
AvrRpm1-F	TGCTGACACGAGTAATCCCA	RT-qPCR validation
AvrRpm1-R	TGATCTGTCATGAGTGCGGT	RT-qPCR validation
Alg8-F	GAGTTCTGTGAAGTGCGTG	RT-qPCR validation
Alg8-R	GCCATCGAGCACATGTTGAT	RT-qPCR validation
Alg44-F	CTGTACTTCGTCAGCCATGC	RT-qPCR validation
Alg44-R	CTTTGCACGGTACCTTCACG	RT-qPCR validation

a Rif^r^, rifampin resistance; Gm^r^, gentamicin resistance; Tc^r^, tetracycline resistance; Kn^r^, kanamycin resistance.

### Construction of c-di-GMP reporters.

To report the c-di-GMP level in P. syringae sensitively, three c-di-GMP reporters of P. syringae pv. syringae B728a were constructed by inserting the promoters of Psyr_0610 (315 bp), Psyr_0685 (257 bp), Psyr_5026 (247 bp) to the promoterless plasmid pMS402 (46) (Table 1). Furthermore, to examine whether their homogenous genes in P. syringae pv. tomato DC3000 are also sensitive to the c-di-GMP level in the P. syringae pv. syringae B728a strain, we constructed the corresponding homogenous promoters originating from P. syringae pv. tomato DC3000 (Table 1). The constructed plasmids were electrotransformed into P. syringae pv. syringae B728a and its derivatives (the high-c-di-GMP-content OX-*yedQ* strain and low-c-di-GMP-level OX-*yhjH* strain) by using a MicroPulser (Bio-Rad) with 1.8 kV measurement during every electroporation. The OX-*yedQ* and OX-*yhjH* strains contain plasmids pBBR1MCS-5 and pBBR1MCS-3, respectively. Positive reporter strains were cultured at mid-log-growth phase (optical density at 600 nm [OD_600_], 0.6). Luminescence values (in counts per second [cps]) of bacteria were recorded using a 96-well white opaque microplate in a BioTek microplate reader with fiber-optic type luminescence. The OD_600_ of bacteria in each well was determined immediately using a 96-well transparent-bottom cell culture plate with the Fisher Scientific microplate reader.

### RNA-seq analysis.

To test the effect of high c-di-GMP levels in P. syringae pv. syringae B728a on the transcriptome, cells were recovered and cultured to early stationary phase (OD_600_, ∼2, with no addition of antibiotics) in NYGB medium consisting of 8 g/liter nutrient broth, 10 g/liter glucose, and 5 g/liter yeast extract. Cells were mixed with bacterial RNAprotect reagent (Qiagen) to keep RNA intact. Total RNA was purified using the RNeasy minikit (Qiagen). The RNase-free DNase set (Qiagen) was applied to remove contaminant DNA through on-column DNase digestion. A second DNA removal step was applied using the Ambion Turbo DNA-free kit. rRNA was depleted using the Ribo-Zero rRNA removal kit (Illumina). The integrity and concentration of total RNA and DNA contamination were examined using the Agilent TapeStation system (Agilent Technologies) and Qubit 2.0 fluorometer (Invitrogen). Reverse transcription into cDNA was done using the NEBNext RNA first- and second-strand synthesis modules (NEB). Analysis of gene expression was carried out via Illumina RNA-seq. Libraries were produced using an Illumina TruSeq stranded mRNA sample prep kit. The libraries were sequenced using an Illumina HiSeq 2500 platform with a paired-end protocol and read lengths of 101 nucleotides (nt). The raw sequence data were streamlined using the Trim Galore! version 0.4.5 software (http://www.bioinformatics.babraham.ac.uk/projects/trim_galore/) to truncate or filter reads of low quality (parameters, –paired -q 20 –phred33 –illumina –length 36). High-quality reads were then aligned to the P. syringae pv. syringae B728a reference genome (GenBank accession no. NC_007005.1) and annotation file (ASM1224v1) using Tophat version 2.2.6 (47) with the parameter “-N 2 -g 1.” Only the reads mapped once were considered. From the resulting alignments, SAMtools version 1.6 (48) was applied to sort the bam file. The differentially expressed genes were identified by performing Cuffdiff version 2.2.1 (49) with a *P* value smaller than 1e−5. Each sample in the RNA-seq assay was repeated three times.

### Reverse transcription-quantitative PCR.

RT-qPCR was done using the OX-*yedQ* and OX-*yhjH* strains to validate the RNA-seq data. Briefly, 500 μl fresh and overnight bacterial cells (OD_600_, 1) was harvested by centrifugation at 2,400 × *g* for 5 min at 4°C. The extraction of total RNA followed the manufacturer’s instructions for the total RNA purification kit (Sangon Biotech). cDNA was synthesized using the TIANScript reverse transcriptase (RT) kit (Tiangen). RT-qPCR used SuperReal PreMix Plus (Tiangen). The mass of cDNA is 70 ng per reaction, and 16S rRNA was selected as the internal reference. Key genes involved in T3SS and biofilm were analyzed. mRNA expression was evaluated for each sample using the cycle threshold (*C_T_*) value. Relative gene expression was calculated as follows: Δ*C_T_* = Δ*C_T_*_target_ − Δ*C_T_*_16S rRNA_. The fold change for the treatment was defined as the relative expression compared with the control group and was calculated by the 2^−ΔΔ^*^CT^* method, where ΔΔ*C_T_* = *C_T_*_control_ − Δ*C_T_* (50, 51). The error bar was calculated using the standard deviation (SD) value. The experiment was repeated three times.

### Quantification of c-di-GMP extracted from P. syringae pv. syringae B728a using liquid chromatography-mass spectroscopy.

P. syringae pv. syringae B728a cells were recovered on a KB agar plate. Single colonies were picked and grew overnight in KB medium with respective antibiotics for strains carrying plasmid (30 μg/ml tetracycline and 30 μg/ml gentamicin) and no antibiotics for the wild type at 28°C and 200 rpm. Overnight cultures were diluted to an OD_600_ of ∼0.01 in KB medium and grew for 16 h to stationary phase (OD_600_ ∼3 for WT and 1.3 to ∼1.6 for the two mutants). Biological triplicates were used for each strain. Five milliliters of cells was collected from each sample and washed with 1 mM ammonium acetate. c-di-GMP was extracted using lysis buffer consisting of ammonium acetate-methanol-water in a 40%/40%/20% ratio. Sonication using a probe sonicator was done using 40% amplitude for one-and-a-half minutes in a 10-s on/10-s off cycle to obtain better cell lysis. Cell debris was removed by centrifugation, and supernatants containing c-di-GMP were dried to remove lysis buffer using a Labconco SpeedVac concentrator. All processing steps were performed on ice or at 4°C. Dried samples were reconstituted in 200 μl of LC-MS-grade water and injected into LC-MS directly with an injection volume of 5 μl. A Waters BEH C_18_ column (1.7 μm, 2.1 by 50 mm) was used for high-performance liquid chromatography (HPLC). The gradient run was applied using water containing 10 mM ammonium formate and 0.1% formic acid as mobile phase A, and methanol containing 0.1% formic acid as mobile phase B. Mobile phase A had a gradient of 100% at 1 min, 80% at 3 min, 60% from 4 to 4.5 min, and 100% from 4.6 to 6 min, applied at a flow rate of 0.3 ml/min. A Xevo TQ-S (Waters) mass spectrometer was used to identify c-di-GMP with reference to c-di-GMP standard (Sigma) using electrospray ionization (ESI)-positive mode at 40-V collision energy. Three technical replicates were measured for each sample. c-di-GMP was detected at a retention time of 1.74 min with multiple reaction monitoring (MRM) transition to 152.0 and 135.0. Quantities of c-di-GMP were calculated and normalized against protein levels and illustrated in Fig. 1. The c-di-GMP levels in the OX-*yhjH* samples were undetectable.

Protein extraction was done for normalizing c-di-GMP concentrations among samples. One milliliter of bacterial culture was collected from each sample at the beginning of extraction from the same sample tubes. The cell pellet was collected and resuspended in 1 ml of 0.1 M NaOH. Cells were heated at 95°C for 10 min. The total protein content of each sample was measured using a Qubit protein assay kit (Invitrogen).

### GO and KEGG pathway enrichment analysis.

GO enrichment analysis and KEGG pathway enrichment analysis of DEGs were performed using DAVID 6.8 online analysis (https://david.ncifcrf.gov/gene2gene.jsp). GO and KEGG terms with a *P* value of <0.05 were defined as a significantly enriched term.

### Congo red assay and biofilm formation assay.

A Congo red agar plate assay was carried out according to the previous study (21), with minor changes to compare the production of exopolysaccharide between the elevated-c-di-GMP-level OX-*yedQ* strain and the OX-*yhjH* strain. Germ-filtered Congo red dye (100 μg/μl) was added to KB medium with 1.5% agar. Five microliters of liquid overnight bacterial culture was spotted to the Congo red plate (with 25 μg/ml rifampin) and cultured at 28°C. The colony staining was photographed after 3 days. The experiment was repeated with two independent bacterial cultures (3 plates for each strain).

A biofilm detection experiment was performed with minor modifications, as reported previously (52). In brief, overnight bacterial culture was transferred to a 10-ml borosilicate tube containing 2 ml KB medium (without antibiotics) at an OD_600_ of 0.1 and cultured statically at 28°C for 3 to 4 days. Crystal violet (0.1%) was used to stain those biofilm cells that adhered to the tube tightly for 15 min, and other cells that bound to tube loosely were washed off with distilled deionized water (ddH_2_O). The tubes were washed gently three times with ddH_2_O, and the remaining crystal violet was fully dissolved in 250 μl of 95% ethanol with constant shaking after photographs were taken. Two hundred microliters of this eluate was transferred to a clear and transparent 96-well plate to measure its optical density at 590 nm using a BioTek microplate reader. Three tubes were used for each strain, and the experiment was repeated with three independent bacterial cultures.

### Measurement of H_2_O_2_ resistance.

For the growth assay, an overnight bacterial culture (OX-*yedQ* and OX-*yhjH* strains; OD_600_, 1.0) was diluted to OD_600_ of ∼0.01 with KB medium (without antibiotics) with or without 2 mM H_2_O_2_. Two hundred microliters of diluted culture medium was added to a sterile 96-well plate (Thermo Fisher Scientific) at 28°C. After 12 h, 24 h, 36 h, and 42 h, bacterial growth at 600 nm was recorded using a BioTek microplate reader. The experiment was repeated with two independent bacterial cultures, and 3 replicates were used for each strain. As described previously (53), in the hydrogen peroxide resistance colony assay, the bacterial culture was adjusted to an OD_600_ of 1.0. KB agar plates were supplemented with 0.5 mM H_2_O_2_, and 10 μl of serially diluted bacterial cultures was incubated on the plates at 28°C for 3 days. Three plates were used for two strains, and the experiment was repeated with three independent bacterial cultures.

### CAS agar assay for iron uptake.

A chrome azurol S (CAS) agar assay was carried out as previously described (24). To make a 100-ml CAS stock solution, 60.5 mg CAS powder (Sigma) was added to 50 ml of ddH_2_O, followed by 10 ml of 1 mM FeCl_3_. Then, 72.9 mg hexadecyltrimethylammonium bromide (HDTMA; Sigma) was fully dissolved in 40 ml ddH_2_O. At last, the entire HDTMA solution (40 ml) was slowly poured into 60 ml of CAS solution with constantly shaking to form a 100-ml CAS stock solution. A CAS agar plate was prepared by 9 parts freshly autoclaved 1.5% agar KB plate and 1 part CAS stock solution. After the agar solidified, two circular holes were dug into CAS agar by the round end of a 1-ml sterile pipette tip. About 2 μl overnight bacterial culture (OX-*yedQ* and OX-*yhjH* strains; OD_600_, 1.0) of the experiment group and control group was added into one of two holes and cultivated at 28°C. The CAS plate (without antibiotics) was photographed after 3 days against a white background. Three CAS plates were used for two strains, and the experiment was repeated with three independent bacterial cultures.

### Motility assay.

The motility experiment was performed based on a previous study (52), with minor modifications. Swimming and swarming ability were tested on a KB plate with 0.3% and 0.4% agar (MP Biomedicals), respectively. Overnight KB cultures were inoculated and adjusted to the same bacterial density (OX-*yedQ* and OX-*yhjH* strains; OD_600_, 1.0). Two-microliter aliquots were dotted on swimming and swarming plates and cultured at 28°C. To quantify bacterial motility, the diameter of each colony was determined. Photographs were taken after 36 h for the swimming plate and 72 h for the swarming plate. Three motility plates were used for two strains, and the experiment was repeated with three independent bacterial cultures.

### Statistical analysis.

Data are presented as the mean ± standard deviation. Data were tested for normality and analyzed using unpaired Student’s *t* test. In Fig. 1 and 3 to 8, one asterisk (*) indicates a significant difference with a *P* value of <0.05. Two asterisks (**) indicate a significant difference with a *P* value of <0.01, and three asterisks (***) indicate a significant difference with a *P* value of <0.001. Each experiment was performed three times with similar results.

### Data availability.

The RNA-seq data sets have been deposited in the National Center for Biotechnology Information (NCBI) database with an accession number GSE120889.

## Supplementary Material

Supplemental file 1

Supplemental file 2
