# Supplementary material for: Pleiotropic Effects of c-di-GMP Content in Pseudomonas syringae
Source: Appl Environ Microbiol. 2019 May 2;85(10):e00152-19. doi: 10.1128/AEM.00152-19 (PMC6498148; doi:10.1128/AEM.00152-19)
Supplement: Supplemental file 1 [file AEM.00152-19-s0001.pdf]

The Pleiotropic Effects of c-di-GMP Content in *Pseudomonas syringae*

Tingting Wang[1†], Zhao Cai[2†], Xiaolong Shao[3], Weitong Zhang[1], Yingpeng Xie[1], Yingchao Zhang[3], Canfeng Hua[1], Stephan C. Schuster[2,5], Liang Yang[2,4], Xin Deng[1*]

Supplemental information

Legend to supplemental figure

Figure S1. GO enrichment analysis in "Biological Process" of RNA-seq between OX-*yedQ* and WT strains

The GO pathways were summarized in three main categories: phosphorelay signal transduction, flagellum-dependent cell motility, and bacterial chemotaxis. The x-axis indicated the numbers of genes of the GO metabolic pathways. The y-axis indicated terms of GO metabolic pathways. GO terms were over-represented by > 2-fold enrichment values, with p value < 0.05.

Table S1. RNA-seq result by using OX-*yhjH* and OX-*yedQ*.

818 differentially expressed genes (DEGs) with q value < 0.05 were defined as significant difference. Two duplicates were carried in RNA-seq data.

Table S2. RNA-seq result of OX-*yedQ* and wild-type.

DEGs were defined with q value < 0.05. Two duplicates were used in RNA-seq data.

Figure S1

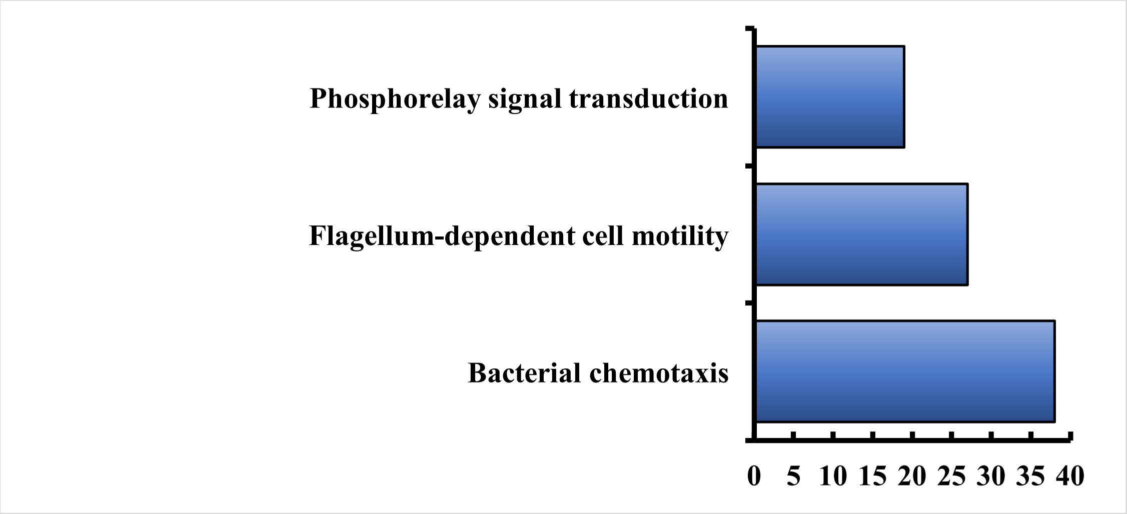
Phosphorelay signal transduction
Flagellum-dependent cell motility
Bacterial chemotaxis
0
5
10
15
20
25
30
35
40
